# Computed Tomography‐Verified Pacing Location of Micra Leadless Pacemakers and Characteristics of Paced Electrocardiograms in Bradycardia Patients

**DOI:** 10.1002/clc.70209

**Published:** 2025-10-07

**Authors:** Jianghua Zhang, Xianhui Zhou, Yanmei Lu, Yaodong Li, Qiang Xing, Zukela Tuerhong, Xu Yang, Jiasuoer Xiaokereti, Yankai Guo, Xiaohong Zhou, Samantha Kohnle, Siyuan Zou, Baopeng Tang

**Affiliations:** ^1^ Cardiac Pacing and Electrophysiology Department The First Affiliated Hospital of Xinjiang Medical University Urumqi China; ^2^ Xinjiang Key Laboratory of Cardiac Electrophysiology and Cardiac Remodelling The First Affiliated Hospital of Xinjiang Medical University Urumqi China; ^3^ Research and Technology Division, Cardiac Rhythm Management, Medtronic Inc. Minneapolis Minnesota USA; ^4^ Cardiac Rhythm Management, Medtronic (Shanghai) Shanghai China

**Keywords:** bradycardia, cardiac computed tomography, leadless pacemaker, right ventricular septum

## Abstract

**Introduction:**

The leadless pacemakers are implanted routinely under fluoroscopic image, yet the pacing sites and corresponding paced electrocardiography (ECG) remain unclear. This study was to determine the computed tomography (CT)‐verified location of the leadless Micra pacemakers (Micra) and ECG characteristics.

**Methods:**

Twenty consecutive patients who met the pacemaker indications for bradycardia and underwent fluoroscopy assisted Micra implantation were enrolled. All subjects underwent a postoperative CT scan to determine the precise location of the Micra pacing tip. Paced 12‐lead ECG characteristics were analysed and correlated with the Micra tip location.

**Results:**

In the nine partitions of fluoroscopic RAO images, 14 (70%) of 20 patients had the Micra tip in zone 5, 5 (25%) in zone 6 and 1 in zone 2. Reconstructed CT 3‐D cardiac images found Micra tips mostly clustered near the anterior insertion between the RV septum and free wall with 12 cases at the insertion‐septal side and 8 at the free‐wall side. ECG morphological analysis found that the peak deflection index in ECG lead V1 was 0.409 ± 0.058 for Micra tips at the insertion‐septal side and 0.527 ± 0.062 in the free‐wall side (*p* < 0.001 between two sides) and *R* wave amplitude in lead V6 appeared larger for Micra tips in the free‐wall group compared to Micra tips in the insertion‐septal group, while there was no difference in QRS duration between two sides.

**Conclusion:**

In routine Micra implantation, the pacing sites were often located in the anterior insertion region.

## Graphical abstract

The CT‐verified locations of the implanted Micra leadless pacemakers clustered near the RV anterior insertion. There were pacing location‐associated characteristics of 12‐lead ECG, suggesting the influence of pacing location on the activation propagation in the ventricles.

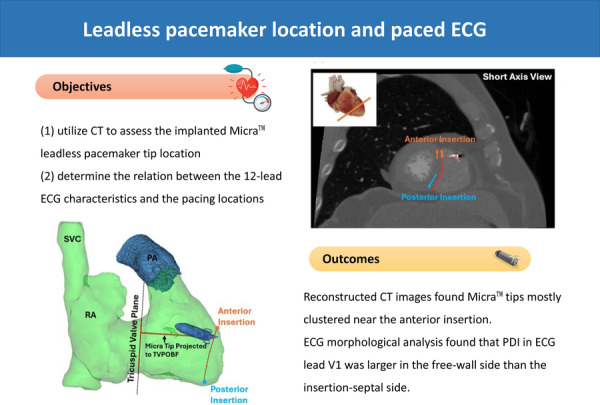

## 1 Introduction

The implantation of conventional transvenous pacemaker, an effective therapy in patients with symptomatic bradycardia, often has implantation‐related complications, including electrode dislocation, electrode fracture, venous thrombosis, tricuspid regurgitation, and, in particular, infection that usually requires a complete removal of the entire pacemaker system [1, 2]. Recently, multiple clinical investigations have demonstrated that leadless pacemakers have lower implantation‐related complications while maintaining its safety and efficacy [3, 4]. Moreover, to avoid the deployment of leadless pacemaker at the RV apex where the procedure could cause epicardial perforation, septal implantation of leadless pacemakers has become a popular choice by implanters [5, 6]. However, the actual pacing position of the conventional fluoroscopy guided leadless pacemaker often remain uncertain due to patients’ heart variations, implant tool limitations, and operators’ preference and experience in clinical practice, which likely leads to inconclusive procedural and clinical endpoints.

The objectives of the present study were (1) to utilize the post‐implant computed tomography (CT) to assess the precise positions of the pacing tip of the implanted Micra leadless pacemaker and compare the CT‐verified pacing sites with the pacing sites determined under the fluoroscopic imaging at the implantation; and (2) to characterize 12‐lead ECG and determine the relation between the 12‐lead ECG characteristics and the pacing locations.

## 2 Methods

### 2.1 Patients

A total of 20 consecutive patients who met Class I or IIa indications for pacemaker therapy and intended to receive Micra leadless pacemaker (Micra) implantation in the RV mid‐septum were prospectively enrolled from February 2021 to July 2023. The exclusion criteria were as follows: (1) presence of thrombosis or cancer embolus in the inferior vena cava (IVC) or portal vein; (2) presence of bilateral femoral vein stenosis or tortuosity that could cause failure to accommodate the Micra; (3) inability to tolerate heparinization or allergy to heparin; (4) presence of implanted devices that interfered with the Micra, such as IVC filters or tricuspid mechanical valve replacement; (5) presence of severe renal insufficiency (glomerular filtration rate < 30 mL/min) or iodine allergy; (6) acute phase of myocardial infarction, and (7) contraindications for cardiac CT scan or contrast agents. All patients signed an informed consent form before the surgical procedure. This study was approved by the Ethics Committee of the First Affiliated Hospital of Xinjiang Medical University and registered in China Clinical Trial Registry. The data that support the findings of this study are available from the corresponding author on reasonable request.

### 2.2 Implantation of Micra Leadless Pacemaker

**Figure 1:**
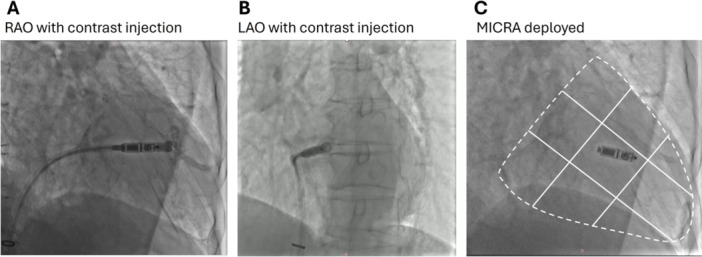
Fluoroscopy‐assisted implantation of Micra leadless pacemaker. (A): Fluoroscopic view with RAO 30; (B): Fluoroscopic view of LAO 45, (C): The Micra tip in zone 5 of the nine‐partition method.

The implantation procedure for Micra has been previously described. Patients were placed in a supine position and local anaesthesia was administered at the right inguinal area for access of the femoral vein through which the Micra delivery catheter (Medtronic, Minneapolis, MN, USA) was advanced into the inferior right atrium via the IVC. The RV septum was visualized via the dye injection of 20 cc contrast through the delivery catheter under fluoroscopic views in the right anterior oblique (RAO) 30° ± 10° (Figure 1A). The site of the Micra was initially targeted in zone 5 of the RV septum based on the 9‐partition method for the zones in the RV septum (Figure 1C). The chosen position for the Micra deployment was further confirmed with fluoroscopic left anterior oblique (LAO) 45° ± 10° positions (Figure 1B). The angle between the Micra TPS and the spinal vertebrates was 70° to 90° and the Micra‐TPS positioning could be confirmed by fluoroscopic imaging of LAO view and the height of spinal vertebrate, which might aid the anchoring of the Micra to the RV septum [7].

If the pacing parameters at the chosen site were unacceptable, the distal delivery catheter was moved to a nearby location. After release of the Micra was completed, electrical testing of the Micra, including pacing capture threshold, pacing impedance, and *R*‐wave amplitude, was conducted to confirm the required capture threshold ≤ 1.5 V, the pacing impedance ranging from 400 to 1500 Ω, and *R*‐wave amplitude ≥ 5 mV. After satisfactory pacing parameters, the pull test verified that at least two tines were fixed in the myocardium, then the tether was cut after pull and hold test and the delivery system was withdrawn, and the puncture site was sutured for haemostasis.

## 3 Data Collection

### 3.1 Baseline Data

Patients’ demographic characteristics and indications for pacemaker implantation were collected at enrollment. The echocardiography parameters measured at enrollment included left ventricular ejection fraction (LVEF) and left ventricular dimensions.

### 3.2 Implantation Data

Pacing parameters including capture threshold, *R*‐wave amplitude, and pacing impedance were recorded at implantation. Paced 12‐lead (ECG) was analysed for QRS duration, left ventricular activation time (the interval from pacing artifact to the peak of ECG V5 or V6), *R*‐wave amplitude in ECG leads I and V6, and peak deflection index (peak deflection index was calculated by dividing the time from the onset to the peak deflection of the QRS by the duration of the QRS [8]). Left ventricular activation time is usually considered to be associated with left ventricular (LV) electrical synchrony, while *R*‐wave amplitude in lead I is associated with activation propagation patterns during free wall pacing [9]. There were 3 electrophysiology physicians involved in obtaining the ECG measurements, each patient's ECG data was measured manually, and the final measurements were made with a consensus between two readers. ECG data was collected by MedEx (MECG‐300). All the individuals who conducted the ECG analysis were blinded to CT and fluoroscopic position of the Micra pacemaker.

Fluoroscopic imaging was collected with RAO 30° ± 10° and LAO 45° ± 10°. The postoperative analysis of the pacing site of each subject with the nine‐partition method of the fluoroscopic RAO view was performed according to the previous description of classifying RV septal regions [10]. As shown in Figure (1C), the border of the ventricles in RAO 30° view was drawn and divided into nine quadrants with the “3 × 3 partitions” from zones 1 to 9. The zone location of the Micra pacing tip was then determined.

### 3.3 Post‐Implant CT Image Processing

**Figure 2:**
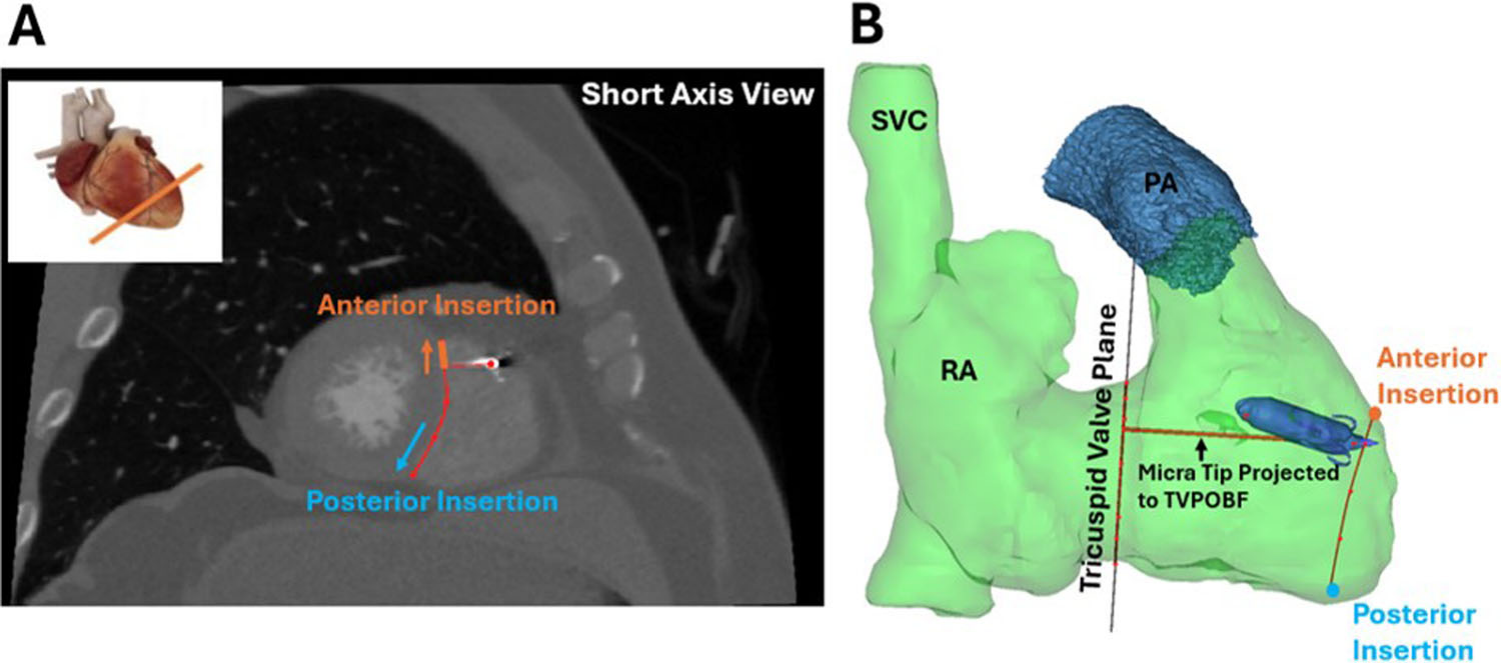
CT image processing to determine the tip location of Micra leadless pacemaker. (A): Short axis CT slice at the Micra tip location showing the distance from the projected Micra tip onto the septum to the anterior insertion (orange). (B): 3D representation of the right ventricle displaying the Micra tip distance onto the TVPOBF.

**Figure 3:**
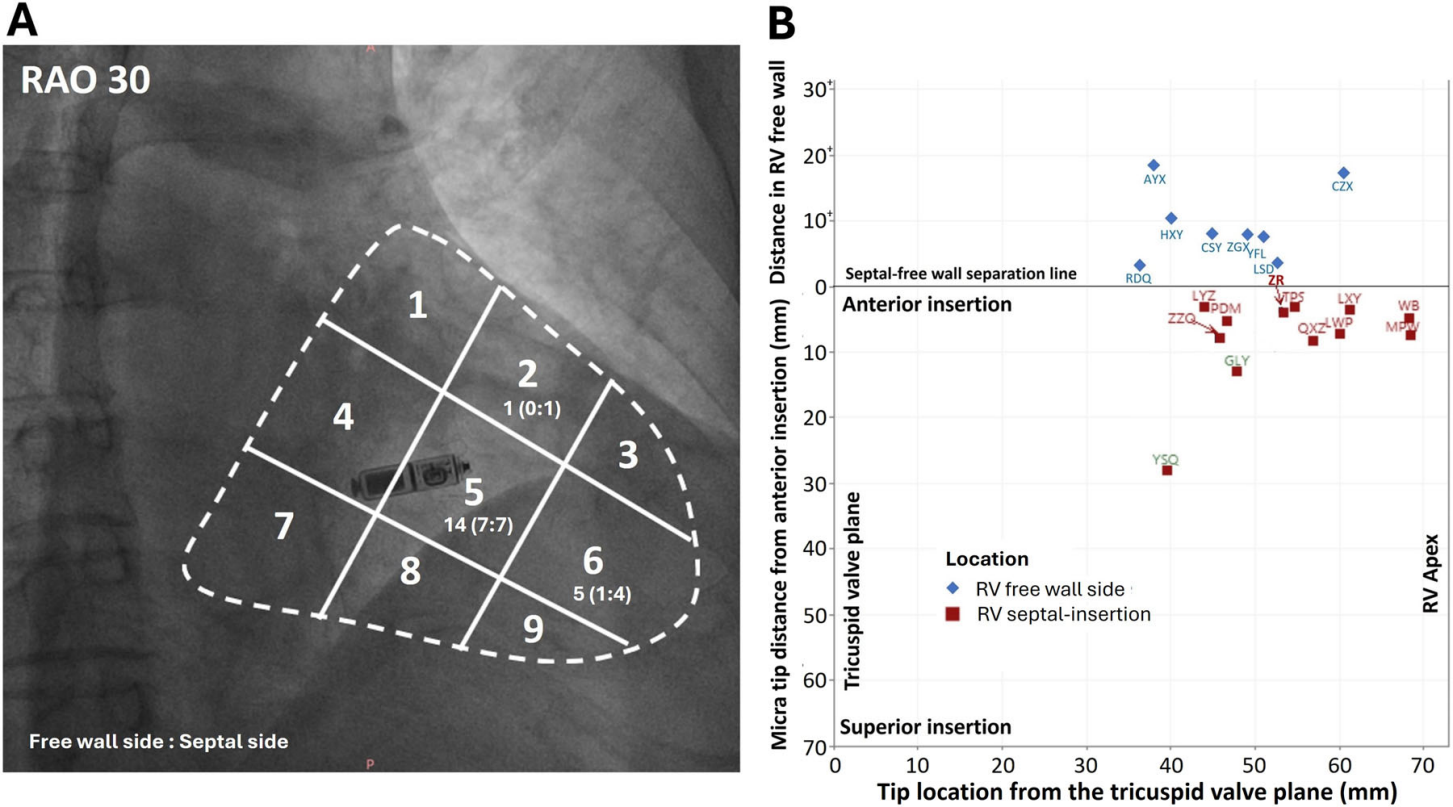
Distribution of Micra tip locations under fluoroscopic RAO view and 3‐D CT image. (A): Fluoroscopic RAO view with nine‐partition grids. Small numbers represent the Micra tip locations in the corresponding zones. (B): Micra tip locations in the CT analysis, with the distance of the Micra tips to the landmark of the anterior towards the superior insertion (*Y*‐axis) and the distance from the tricuspid valve plane towards the RV apex (*X*‐axis), RV apex, and tricuspid valve plane, with 12 Micra tips in the RV septal‐insertion region and 8 in the free‐wall side.

All patients underwent a computed tomography (CT) scan of the heart with contrast enhancement during the peri‐implant period (Day 2 to Day 5 post implantation). Cardiac CT imaging is a reliable method to identify the location of a pacing lead [11]. CT imaging data was processed as described previously using the software Materialise Mimics software (Version 22, Materialise, Leuven, Belgium) [12]. Image sets were assessed for quality and the best diastolic and best systolic image phases were selected for analysis. Various threshold masks were applied to the images to select structures of interest. Three‐dimensional models (Figure 2) were created from the segmentation masks for the right atrium (RA), right ventricle (RV), pulmonary artery (PA), left ventricle (LV), aortic root (AoR), and the implanted Micra. In a short axis left heart view, the Micra tip was located (Figure 2A). The tip location was viewed in both the 2D left heart short axis view (Figures 2A) and 3D segmentation viewer (Figure 2B) to approximate whether the tip was located on the septum, anterior insertion, or the RV free wall. To describe the size of the septum, the septal longitudinal length from the centroid of the tricuspid valve to the RV apex was measured. To determine the depth of the Micra tip location, a spline was defined with the visualization of the tricuspid valve (TV) annulus, then the Materialise Mimics software planarized and compressed the three‐dimensional TV spline points into a two‐dimensional depiction of the TV plane of best fit (TVPOBF) [12]. The tricuspid valve plane of best fit (TVPOBF) was created and the Micra tip was projected onto the plane using the shortest distance (Figure 2B). The Micra tip location was described in multiple ways (Figure 2): (1) the distance from the Micra tip to the TVPOBF, (2) the distance from the Micra tip to the RV apex, (3) the distance from the Micra tip site within the RV septum to the RV anterior insertion, and (4) the distance from the Micra tip site within the RV free wall to the RV anterior insertion (Figure 3B). All CT‐image measurements were performed during the end diastolic and systolic phases.

### 3.4 Follow‐Up

All patients underwent regular device follow‐up at a hospital device clinical office at 3‐month post implantation. Pacing parameters were checked. Adverse events were documented, including arteriovenous fistula, hematoma, incision site bleeding, persistent lymphatic fistula, vascular pseudoaneurysm, and other complications such as pericardial effusion and perforation, as well as leadless pacemaker displacement and embolization.

### 3.5 Statistical Analysis

Shapiro‐Wilk test was used to confirm the data in the parametric distribution with statistical significance > 0.05. Continuous numerical variables are presented as mean ± standard deviation (SD) and categorical variables as percentages and values. Otherwise, median and interquartile range were used to express data with a skewed distribution. Quantitative data were compared using t‐test. Comparisons between groups were performed with two‐sampled t‐tests. A two‐sided test was used, and statistical significance was set at *p* < 0.05. All statistical analyses were performed using the SPSS 25.0 software.

## 4 Results

### 4.1 Patient Characteristics

All enrolled patients underwent successful implantation of a Micra. Patient baseline characteristics and pacemaker indications were summarized in Table 1. Of 20 patients, 9 (45%) had sinus node dysfunction, 7 (35%) had II‐III degrees of atrioventricular conduction block, 4 (20%) had sinus node dysfunction with AV conduction abnormality, and 4 (20%) had a history of atrial fibrillation. Overall, baseline LVEF and LVEDD were normal (Table 1). The baseline intrinsic ECG QRS duration was 97.8 ± 22.3 ms with two patients having right bundle branch block and paced QRSd was 150.6 ± 17.8 ms. No complications were reported at implantation of Micra and 3‐month follow‐up visits.

**Table 1:** Patient characteristics.

Characteristic	All patients (*N* = 20)	Micra in Septum (*N* = 12)	Micra in FW (*N* = 8)	*p*
Age (year)	68.2 ± 17.6	61.4 ± 18.7	78.4 ± 9.4	0.016
Gender (Male) (%)	10 (50.0)	5 (41.7)	5 (62.5)	0.650
Weight (kg)	69.0 ± 10.8	70.6 ± 10.5	66.5 ± 11.4	0.432
BMI (kg/m^2^)	24.5 ± 3.6	25.1 ± 4.0	23.7 ± 2.8	0.377
Hypertension (%)	11 (55.0)	6 (50.0)	5 (62.5)	0.582
Diabetes (%)	7 (35.0)	4 (33.3)	3 (37.5)	0.848
CAD	0	0	0	—
CKD (%)	1 (5.0)	1 (8.3)	0	0.834
NYHA				
III‐IV (%)	4 (20.0)	2 (16.7)	2 (25.0)	1.000
I‐II (%)	16 (80.0)	10 (83.3)	6 (75.0)	0.909
Pacemaker indication				
SND (%)	9 (45.0)	4 (33.3)	5 (62.5)	
AVB‐II (%)	1 (5.0)	1 (8.3)	0	
AVB‐III (%)	6 (30.0)	6 (50.0)	0	
SND + AVB (%)	4 (20.0)	1 (8.3)	3 (37.5)	
AF (%)	4 (20.0)	3 (25.0)	1 (12.5)	
Echocardiography				
LVEF (%)	61.4 ± 3.2	61.4 ± 3.8	61.4 ± 2.1	0.986
LVEDD (mm)	48.9 ± 3.6	47.9 ± 3.9	50.3 ± 2.9	0.144
LVESD (mm)	33.0 ± 2.8	32.5 ± 3.1	33.6 ± 2.4	0.372
12‐lead electrocardiography*				
Intrinsic QRSd (ms)	97.8 ± 22.3	99.0 ± 15.1	97.0 ± 27.5	0.886
LBBB (%)	0	0	0	—
RBBB (%)	2 (20.0)	1 (25.0)	1 (16.7)	0.628
Device interrogation				
Pacing capture threshold (V)	0.59 ± 0.24	0.58 ± 0.26	0.61 ± 0.23	0.741
Paced *R*‐wave amplitude (mV)	0.17 ± 0.18	0.15 ± 0.19	0.18 ± 0.19	0.754
Impedance (Ω)	848.5 ± 162.5	786.6 ± 117.0	941.3 ± 183.8	0.059
Pacing percentage (%)	49.3 ± 36.1	52.7 ± 43.9	44.1 ± 21.3	0.568

*Note:* Values are mean ± SD or *N* (%).

Abbreviations: AVB, atrioventricular block; CAD, coronary artery disease; CKD, chronic kidney disease; FW, free wall; LBBB, left bundle branch block; LVEDD, left ventricular end‐diastolic dimension; LVEF, left ventricular ejection fraction; LVESD, left ventricular end‐systolic dimension; NYHA, New York Heart Association; SND, sinus node dysfunction.

* For 12‐lead electrocardiography, *N* = 10 in all patients, *N* = 4 for Septum group, *N* = 6 in FW group.

### 4.2 Micra Tip Location

At the implantation under fluoroscopic view of RAO and LAO, the Micra was deployed at zone 5 in 14 (70%) of 20 patients, zone 6 in five patients (25%), and zone 2 in 1 patient (5%) (Figure 3A). However, the postimplantation CT image analysis found that the Micra tip locations mostly clustered around the anterior insertion (Figure 3B), with 12 (60%) patients having their Micra tip locations in the RV septum or insertion (the septal‐insertion group) and 8 (40%) in the RV free‐wall side (the free‐wall group) of the insertion line (Figure 3B). In the septal‐insertion group, two patients clearly had their Micra tip in the RV septum (patients GLY and YSQ in Figure 3B). The tip distance to the anterior insertion was 8.0 ± 7.1 mm for Micra tips in the septal‐insertion group and 9.5 ± 5.9 mm for Micra tips in the free‐wall group (Figure 3B).

Of 14 patients with their Micra tip in zone 5 of the fluoroscopic view of RAO, only 7 (50%) had their Micra tip location in the RV septum or anterior insertion in the CT image analysis while the remaining 7 (50%) had their Micra tip location in the RV free‐wall side of the anterior line (Figure 3). Of 5 patients with their Micra tip in zone 6 of the fluoroscopic view of RAO, 4 (80%) had their Micra tip location in the RV septum or anterior insertion in the CT image analysis and one in the RV free‐wall side (Figure 3). The remaining 1 patient had the Micra tip in the RV insertion in both fluoroscopic image and CT image.

During the best diastolic period in the CT analysis, the length from the RV apex to the tricuspid valve annulus centroid was 72.6 ± 9.7 mm with 69.5 ± 10.3 mm for Micra tips in the free‐wall group and 74.7 ± 9.1 for Micra tips in the septal‐insertion group (*p* = 0.266 vs. in the free‐wall side). The distance from the Micra tip to the TVPOBF was 49.1 ± 9.6 mm with 51.9 ± 7.1 mm for Micra tips in the free‐wall group and 47.3 ± 10.9 mm for Micra tips in the septal‐insertion group (*p* = 0.270 vs. in the free‐wall side). There was no significant difference in the distance from the Micra tip to the RV apex between the septal‐insertion group (39.4 ± 10.8) and the free‐wall group (44.1 ± 7.7, *p* = 0.271) (Figure 3B).

### 4.3 Pacing Location‐Based ECG Characteristics

**Figure 4:**
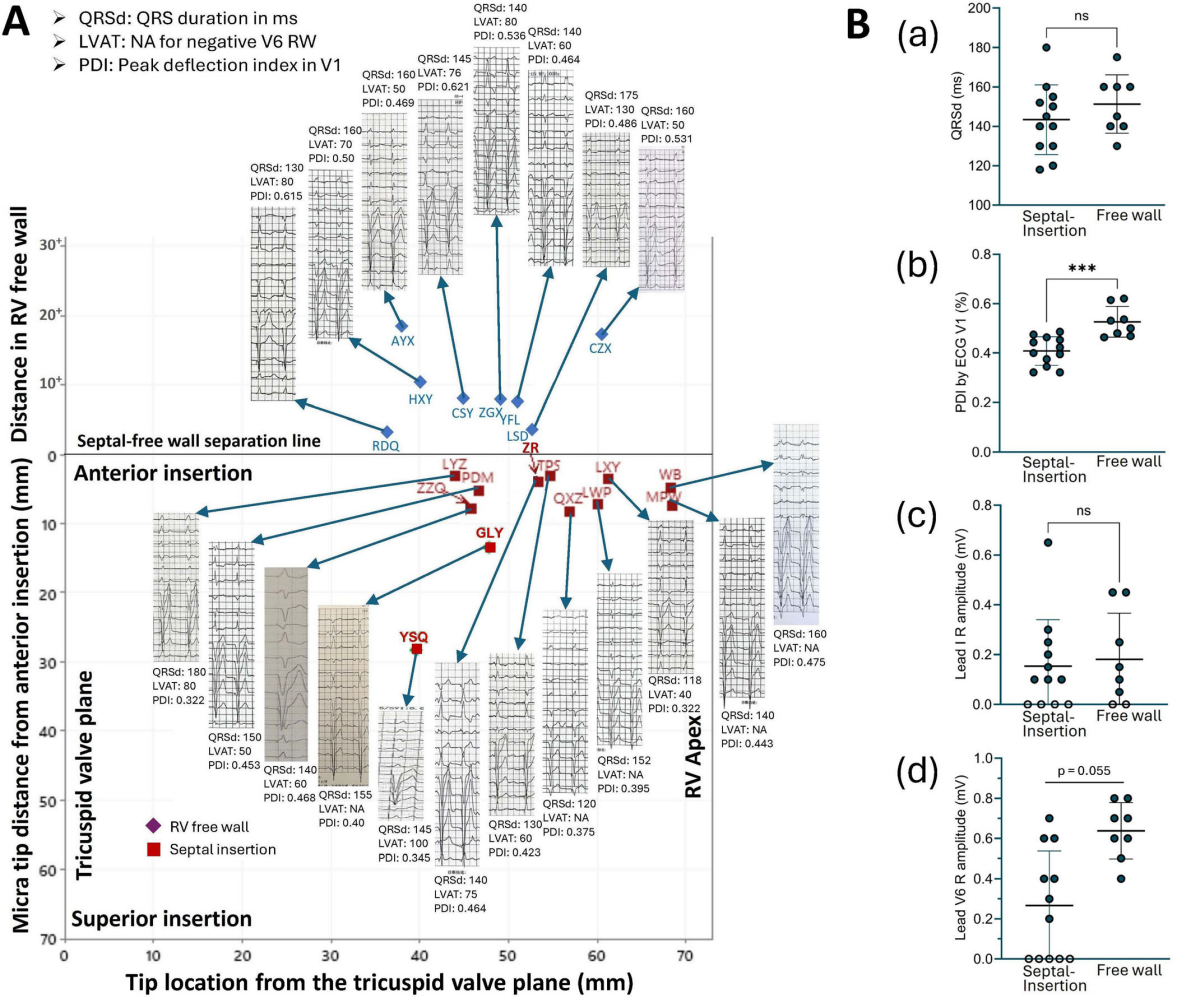
Micra tip location‐based characteristics of 12‐lead ECG. (A) Paced 12‐lead ECG for each corresponding CT‐verified Micra tip location with ECG measurements of paced QRS duration (QRSd), left ventricular activation time (LVAT, 0 if no positive *R* wave in lead V5 or V6), and peak deflectiontime in ECG lead V1 (PDI). (B) individual ECG QRSd (a), PDI (b), lead I *R*‐wave amplitude (c) and V6 *R*‐wave amplitude (d) for Micra tip locations in the septal‐insertion region and free‐wall side of the anterior insertion. Open dots represent no positive values that were not included in the statistical test.

Figure 4 presents the paced 12‐lead ECG and ECG characteristics. The overall QRSd was 146.5 ± 16.7 ms with 151.3 ± 14.8 ms for the free‐wall group and 143.3 ± 17.7 ms for the septal‐insertion group (*p* = 0.295 between two sides) (Figure 4B). The LVAT was 70.7 ± 22.8 ms with 74.5 ± 25.6 in eight cases with Micra tips in the free‐wall group and 66.4 ± 20.1 ms in seven cases with Micra tips in the septal‐insertion group (*p* = 0.507 between two sides) (Figure 4A). *R* wave amplitude in lead V6 was larger for Micra tips in the free‐wall group (0.638 ± 0.141 mV) compared to Micra tips in the septal‐insertion group (0.267 ± 0.271, *p* < 0.001) with no *R* wave amplitude in ECG lead V6 in five patients; the statistical test excluded these five patients showing the same trend in *R* wave amplitude of ECG lead V6 with free‐wall group (0.638 ± 0.141 mV) compared to Micra tips in the septal‐insertion group (0.457 ± 0.181, *p* = 0.055) (Figure 4B). There was no significant difference in the *R* wave amplitude of the ECG lead I between two groups. The peak deflection index in ECG V1 lead was significantly shorter in the septal‐insertion group (0.409 ± 0.058) than in the free‐wall group (0.527 ± 0.062, *p* < 0.001 between two groups) (Figure 4B).

### 4.4 Pacing Parameters

As shown in Table 1, pacing capture threshold and ventricular *R*‐wave sensing amplitude were in the normal range with no significant difference between two groups. The pacing impedance was significantly smaller in the septal‐insertion group (786.6 ± 117.0 Ω) than those in the free‐wall group (941.3 ± 183.8 Ω, *p* = 0.059).

## 5 Discussion

### 5.1 Major Findings

The present study found that (1) the CT‐verified locations of the implanted Micra leadless pacemakers clustered near the RV anterior insertion, either on the RV septal and insertion or RV free wall, while the operator deployed the Micra leadless pacemakers aiming for the RV septum based on the fluoroscopic view; (2) there were pacing location‐associated characteristics of 12‐lead ECG, suggesting the influence of pacing location on the activation propagation in the ventricles; (3) Pacing impedance was significantly lower for Micra tips in the RV septum‐insertion than those in the free wall.

Placement of a Micra leadless pacemaker at the RV septum is performed under fluoroscopic image, and sometimes with the assistance of right ventriculography via contrast injection that shows the RV septum in fluoroscopic RAO view. The target site for RV septal placement of Micra can be further confirmed with LAO view. In the practice of conventional transvenous RV lead placement at the RV septum, multiple investigations have often found unexpected RV lead tips actually at the RV free wall following post‐implant cardiac CT verification of the lead tip location. Even with operators’ intention of placing the transvenous RV lead tip at the RV septum, the chance of ultimate locations in the RV free wall could be as high as 50%–60% [11, 13]. The present study utilizing the advanced CT software identified more precise location of Micra tips than previous reports and found that tips clustered at the RV anterior insertion or nearby RV septum or free wall between the mid‐septum and apex (Figure 3), with 40% Micra tips at the RV free wall side of the anterior insertion. The Micra tips at the RV free wall had a short distance to the anterior insertion. Thus, while the operator might intend to place the Micra tip in zone 5 (an assumed mid‐septum) of the fluoroscopic RAO 30 (Figure 3A), the ultimate locations of Micra tips were often at the region of the RV anterior insertion or free wall, suggesting that the fluoroscopic view may not warrant the Micra tips at the RV mid‐septum and/or there is difficulty in getting a true septal location despite using contrast and orthogonal *X*‐Ray views.

The pattern of ventricular activation propagation is associated with the origin of the activation in the ventricles. For example, pacing at the RV apex can cause the pattern of complete left bundle branch block in 12‐lead ECG [14]. Pacing at RV free wall created a longer QRSd (169 ms on average) and a larger *R* wave amplitude of ECG lead I than pacing at the RV septum (QRSd: 156 ms on average) [9] while others reported a *Q* wave in ECG lead I [15]. Thus, the characteristics of paced ECG sometimes suggest the location of the transvenous RV lead tip in the RV septum. In the present study, *R* wave amplitude of ECG lead I appeared no different between pacing at the RV septal‐insertion region versus pacing at the nearby free wall. However, the present study found that paced *R* wave amplitude was smaller at the RV septal‐insertion region compared to that at the nearby free wall. Furthermore, the present study found no *R* wave in ECG lead V6 in 5 of 12 cases (42%) with Micra tips at the RV septal‐insertion region while all patients (100%) with their Micra tips at the RV free wall had *R* wave in ECG V6. These findings may suggest that smaller paced *R* wave amplitude or no paced *R*‐wave in ECG lead 6 implies a likelihood of pacing site in the septal‐insertion region. Moreover, the PDI, which represents the direction of activation propagation in the ventricles [8], was significantly smaller for Micra tips in the RV septal‐insertion region compared with in the free wall side. This result in present study provides additional information in the correlation with PDI and Micra tip locations for clinical practice. In the present study, QRSd in the septal‐insertion pacing was 143 ms on average, which appeared shorter than that of the free‐wall side pacing (151 ms on average), however, it is not clear whether this 8 ms shortness in QRSd by septal‐insertion pacing would generate a better ventricular synchrony. These findings suggest that ventricular activation propagation might be different between pacing at the RV septal region (including the anterior insertion) versus at the free wall region. However, there was no significant difference in paced QRSd and LVAT between two groups with two different pacing sites, which might be due to most of pacing sites in the present study clustering in a small region of the anterior insertion [16].

Multiple clinical studies have demonstrated that leadless pacemaker has advantages in the implant‐associated complications over traditional transvenous RV lead without compromise in clinical safety and efficacy. While the originally recommended location for leadless pacemakers is the RV apical region, the RV septal region has recently been adopted for reducing the chance of pericardial effusions [17, 18]. The present study did not observe pericardial effusions and other procedural complications even though the Micra leadless pacemaker was implanted at the anterior insertion, septum or free wall. While fluoroscopic image would not be able to fully ensure the accuracy of Micra tips deployed at the RV mid‐septum, ECG characteristics and pacing impedance might provide additional information for improving the chance for Micra tips being deployed at the RV septum as discussed above. Improvements in tools and images are needed to ensure the accuracy of deploying the leadless pacemakers at the target location.

### 5.2 Limitation

The sample size in the present study was small. While the present study found ECG characteristics and pacing impedance associated with CT‐verified Micra tips locations, the small sample size prevented an algorithm from being developed for guiding Micra tip septal deployment. However, the present study can be considered as an initiative with findings for designing future large‐scale studies. The present study found that most of Micra tips were clustered at the anterior insertion, yet the comparison between pacing sides at RV septum versus free wall was retrospective. Furthermore, the present study only reported the procedural endpoint, not a clinical endpoint. Future clinical investigation with long‐term follow‐up is needed to differentiate the clinical outcomes between leadless pacemakers at the RV septum and those at RV free wall.

## 6 Conclusion

In routine Micra implantation with assistance of fluoroscopic imaging for the target location in the RV mid‐septum, the majority of the pacing sites were located in the region of the anterior insertion, or nearby the RV septum and free wall. Improvements in the implant tools and images may facilitate the Micra tip being deployed at the RV septum.

## Ethics Statement

This study was approved by the Ethics Committee of the First Affiliated Hospital of Xinjiang Medical University and registered in China Clinical Trial Registry. This study was performed in line with the principles of the Declaration of Helsinki.

## Consent

All enrolled patients have provided written informed consent.

## Conflicts of Interest

Xiaohong Zhou, Samantha Kohnle and Siyuan Zou are Medtronic employees. The other authors declare no conflicts of interest.

## Data Availability

The data that support the findings of this study are available from the corresponding author upon reasonable request.
